# Secondary Prevention after Myocardial Infarction: What to Do and Where to Do It

**DOI:** 10.31083/j.rcm2306210

**Published:** 2022-06-08

**Authors:** Vladimír Tuka, Josef Holub, Jan Bělohlávek

**Affiliations:** ^1^2nd Department of Medicine - Department of Cardiovascular Medicine, General University Hospital in Prague, First Faculty of Medicine, Charles University, 12808 Prague, Czech Republic

**Keywords:** cardiovascular rehabilitation, lifestyle, myocardial infarction, pharmacotherapy, secondary prevention

## Abstract

Acute myocardial infarction is a manifestation of atherosclerosis which may be fatal. In-hospital and short-term mortality rates after an acute myocardial infarction have declined in the past few decades. However, although long-term mortality has decreased, it remains unacceptably high. This review paper summarises the non-pharmacological interventions (smoking cessation, physical activity, nutrition, and psychosocial intervention) and pharmacological approaches (antiplatelet and lipid-lowering therapy, renin-angiotensin-aldosterone system inhibitors, beta-blockers, and glucose-lowering drugs) to secondary prevention after a myocardial infarction. The provision of secondary prevention services is established through cardiac rehabilitation, which consists of several discussed components. Finally, we discuss the quality indicators for long-term care after an acute myocardial infarction.

## 1. Introduction 

In 2021, cardiovascular diseases (CVD) were still the most common cause of death in Europe [1]. Ischemic heart disease (IHD) and its complications, such as acute myocardial infarction (AMI), are the most common cause of CVD death accounting for 38% of all CVD deaths in females and 44% in males in the European Society of Cardiology (ESC) member countries [1]. Before the COVID-19 pandemic, estimates indicated that IHD accounted for 5.8 million new cases in this region [1].

AMI is defined as “myocardial cell death due to prolonged ischaemia”, with atherosclerotic plaque disruption with thrombosis accounting for most cases [2]. Atherosclerosis is a progressive chronic inflammatory process resulting from the accumulation of fatty material in the intimal part of the vessel wall in response to the biological effects of risk factors [3, 4]. The concept that coronary heart disease and its complications could be prevented was introduced in the 1960s in the first paper from the Framingham study [5]. From that time, cardiovascular prevention has gained its place in the armamentarium of cardiologists. Secondary prevention is targeted at persons with established CVD (e.g., after AMI) to prevent any further events and improve their quality of life.

Due to primary prevention, hospitalisation rates due to AMI have decreased over the past 30 years [6]. Before the advent of modern therapies, in-hospital mortality had reached almost 30%. The introduction of coronary care units, defibrillation, thrombolysis, coronary angioplasty, and antiplatelet therapy have reduced the in-hospital mortality to around 7% [7]. Data from the French Registry of Acute ST-Elevation or Non-ST-Elevation Myocardial Infarction – (FAST-MI Program) from metropolitan France have shown a decrease in in-hospital mortality from 14% to 3% and from 11% to 3% in ST-elevation myocardial infarction (STEMI) and non-ST-elevation myocardial infarction (NSTEMI) patients, respectively. The same registry noted a six-month mortality decrease from 17.2% to 5.3% and from 17.2% to 6.3% in STEMI and NSTEMI patients, respectively [8].

Even if patients survive the first year after AMI, the mortality rates in the following years are high. The ongoing three-year all-cause mortality ranges between 19.6% to 30.2%, and the composite endpoint of AMI, stroke, or death from 26.0% to 36.2% [9].

Although the prognosis of patients after AMI has improved, it is far from optimal, especially when compared with the general population [10]. Patients after AMI have a short-term and long-term residual cardiovascular risk [11]. The EUROASPIRE IV survey has shown a high prevalence of unhealthy lifestyles and inadequate risk factors control despite the reported high use of secondary preventive medication [12].

Our review summarises the current state of secondary prevention and cardiovascular rehabilitation in patients after AMI.

## 2. Lifestyle 

Lifestyle measures are the basis for successful prevention, from primordial to secondary, extending far beyond the cardiovascular system [13]. They stand as the essential secondary prevention therapy in the STEMI and NSTEMI guidelines [14, 15]. In a secondary analysis of the OASIS-5 study, non-adherence to behavioural recommendations (adherence to diet, exercise and smoking cessation) was associated with a 3.8 fold increased risk of major adverse cardiac events (myocardial infarction (MI), stroke, and death) [16].

### 2.1 Nicotine Dependence 

Smoking cessation is of utmost importance as it is associated with an increased risk of recurrent coronary events. Patients who continue to smoke have a 51% higher risk of recurrent coronary events than non-smokers. Nonetheless, patients who quit smoking after AMI have a 17% higher risk of recurrent events than non-smokers, but their risk declines quickly and approaches that of non-smokers within three years after cessation [17].

Electronic cigarettes (e-cigarettes) have emerged over the last few years and have been advertised as a “healthy alternative” to classical tobacco cigarettes. However, some studies have reported an increased risk of MI in persons using e-cigarettes (odds ratio (OR) 1.79) compared to non-smokers, whereas conventional cigarette smoking has an OR 2.72 [18]. Data on e-cigarette use in patients after AMI is lacking. Some small studies indicate that e-cigarettes are harmful due to their effect on the cardiovascular system. Vaping leads to an acute increase in both heart rate and blood pressure [19, 20] and shifts the heart rate variability towards a sympathetic predominance [21].

Some authors have recommended the use of e-cigarettes as a smoking cessation tool. Due to insufficient data, the use of e-cigarettes for smoking cessation is advised by neither the US Preventive Services Task Force (USPSTF) [22] nor the European Association of Preventive Cardiology (EAPC) [23]. The latter recommends considering e-cigarettes to aid tobacco cessation only alongside a formal tobacco cessation programme [23].

### 2.2 Physical Activity and Cardiovascular Fitness

Exercise training in patients with coronary artery disease (post-MI, with angiographically documented coronary artery disease or after coronary intervention) can decrease total mortality by 20% and cardiovascular mortality by 26% [24]. Exercise leads to increased coronary blood flow and higher myocardial oxygen delivery; repeated increases in blood flow improve endothelial and coronary smooth muscle function, leading to improved coronary vasodilatation [25]. Furthermore, exercise has beneficial effects on cardiovascular risk factors [26]. Exercise does not add much to low-density lipoprotein cholesterol (LDL-C) lowering in patients already on statin therapy but changes the LDL-C particles’ structures, increases high-density lipoprotein cholesterol (HDL-C) levels, and lowers triglyceride levels [27, 28]. Aerobic and resistance exercise decreases the averaged systolic blood pressure (SBP) by 2–4 mmHg and diastolic blood pressure (DBP) by three mmHg [29].

### 2.3 Nutrition and Weight Management 

Determining the exact impact of dietary changes on the prognosis of AMI patients is complicated. The general principles of heart-healthy diets also apply to post-acute coronary syndrome (ACS) patients [30]. The Lyon Diet Heart Study has shown that a Mediterranean-type diet reduces the recurrence rate after the first myocardial infarction, and this protective effect lasted for four years [31]. A recent AHA statement on dietary guidance shifts the focus more on dietary patterns, such as the Mediterranean style, the Dietary Approaches to Stop Hypertension (DASH) style, the Healthy US-Style, and healthy vegetarian diets. All these diets improve cardiovascular health [32].

The dietary approach (calory restriction) is also necessary for bodyweight reduction [33]. Overweight and obese patients with an AMI are younger than those with normal body weight and have a higher risk of developing heart failure. However, the overall in-hospital mortality is not increased [34]. Metabolic syndrome is associated with an increased three-years cardiovascular mortality and reinfarction in patients with NSTEMI [35]. Nevertheless, studies directed at bodyweight reduction in post-ACS patients are lacking. There are also no studies focusing on bariatric surgery after ACS, but some evidence suggests that bariatric surgery could help in improving the prognosis. In one study, bariatric surgery prior to the cardiac event had a protective effect on survival after an AMI [36]. In a prospective SOS (Swedish Obese Subjects) study in a non-randomised design, a reduction of cardiovascular death in the bariatric surgery group was observed compared with the control group. Unfortunately, one of the exclusion criteria was MI during the previous six months, and in the whole cohort, only 1.4% of the patients had previous MI [37].

### 2.4 Psychosocial Risk Factors 

Psychosocial risk factors (PSRFs) influence the cardiovascular system through changes in the immune, neuroendocrine, and behavioural pathways [38], while at the same time, CVD leads to patients’ distress [39, 40]. As depression increases the risk of fatal and nonfatal cardiovascular events in post-ACS patients, the American Heart Association (AHA) recommends considering depression as a risk factor for adverse medical outcomes in ACS patients [41]. The screening and treatment of PSRFs are among the core components of cardiac rehabilitation/secondary prevention (CR/SP) programmes [42]. The depression treatment decreases symptoms in coronary patients and improves the heart-related quality of life (HRQoL), but without any proven effect on mortality [43].

Intriguingly, the prevalence of depression remains the same during the first year after an acute coronary event, but the fluctuation of depressive symptoms is considerable in individual subjects. Thus, depressive symptoms disappear without treatment in some patients, whereas in others without depressive symptoms, these appear during the consequent months [44]. Therefore, screening for depressive symptoms is advised during the acute event and during at least the consequent year.

## 3. Risk Factors and Pharmacotherapy

Even though lifestyle measures are essential, pharmacotherapy to the targeted dosages derived from randomised trials is necessary. The primary pharmacotherapy post ACS can be summarised under the acronym BASIC (Table 1). Antiplatelet and lipid-lowering therapies have very few contraindications, and most patients should receive them.

**Table 1.** **BASIC secondary prevention pharmacotherapy after myocardial infarction**.

B	Beta-blockade
A	Aspirin – low-dose
S	Statin or other hypolipidemic agents
I	Inhibitors of the RAAS system (ACE inhibitors, ARBs = angiotensin receptor blockers)
C	Clopidogrel (or newer ${\mathrm{P2Y}}_{12}$ receptor inhibitors)

RAAS, renin-angiotensin-aldosterone system; ACE, angiotensin converting enzyme; ARB, angiotensin receptor blocker.

### 3.1 Antiplatelet Therapy

Platelet activation plays a central role in atherothrombosis development, and therefore antiplatelet therapy is an integral part of cardiovascular disease prevention and treatment [45]. The duration and type of antiplatelet therapy depends on an individualized assessment of the ischemic and bleeding risks. After an ACS event, dual antiplatelet therapy (DAPT) is indicated for 12 months unless contraindicated [15]. Acetylsalicylic acid (ASA) (at doses of 75–100 mg s.i.d.) remains the mainstay of DAPT, and a potent ${\mathrm{P2Y}}_{12}$ receptor inhibitor is added (prasugrel 10 mg s.i.d., ticagrelor 90 mg b.i.d. or clopidogrel 75 mg s.i.d.). In most patients, ticagrelor or prasugresl are preferred, and clopidogrel should be used when modern ${\mathrm{P2Y}}_{12}$ inhibitors are unavailable or contraindicated. The duration of DAPT can be shortened or extended depending on the balance between the bleeding and thrombotic/ischemic risks. In patients with high and very high bleeding risk, the shortening of DAPT to six months can be considered, and antiplatelet treatment with only ASA, (eventually clopidogrel) continued. In patients with low bleeding risk and high ischemic risk who have tolerated 12 months of DAPT, prolongation of DAPT, e.g., ASA with ticagrelor 60 mg b.i.d. for more than one year (up to three years), may be considered. A combination of ASA and low-dose rivaroxaban (2.5 mg b.i.d.) may also be considered in these patients, especially in the presence of diabetes), chronic kidney disease or peripheral arterial disease [46]. The concomitant use of anticoagulation therapy also influences the duration and type of antiplatelet therapy. The proper timing and extent of antithrombotic therapy are out of the scope of the current review; more information can be found in the guidelines and position papers of the ESC [15, 46].

### 3.2 Lipid-lowering Therapy

The first publication of the Framingham Study already noted that high blood pressure and high cholesterol levels were associated with increased incidence of IHD and AMI [5]. After which, evidence has emerged about the beneficial prognostic effect of lipid-lowering therapy. After ACS, both the magnitude and speed of achieving recommended cholesterol levels are essential. Data from the SWEDEHEART registry show that more significant early low-density lipoprotein cholesterol (LDL-C) reductions are associated with a reduced hazard ratio (HR) of all-cause mortality (HR 0.71) and CV mortality (HR 0.68). Moreover, patients benefited most from high-intensity statin therapy [47]. In the ALPS-AMI study, rapid LDL-C reduction to target levels within four weeks after the acute event was associated with better outcomes in patients after AMI [48].

Nevertheless, statin therapy does not eliminate cardiovascular risk [49], and some patients on maximal statin therapy do not meet the guideline-recommended levels of LDL-C (<1.4 mmol/L in post-ACS patients) [50]. In such cases, the addition of other lipid-lowering agents is indicated. The first choice is ezetimibe, especially in patients with LDL-C levels near the target levels. Adding ezetimibe to statin therapy can lead to a mean LDL-C decrease of 16.7 mg/dL (0.43 mmol/L), i.e., a further 24 % LDL-C level lowering [51].

When the LDL-C is high despite high dose statin therapy, the addition of PCSK-9 inhibitors is an option. In ODYSSEY OUTCOMES, the addition of alirocumab in post-ACS patients resulted in a mean LDL-C of 1.2 mmol/L in the treatment group compared to 2.5 mmol/L in the placebo group. This difference was accompanied by a lower risk of recurrent ischemic cardiovascular events (HR 0.85) [52]. Furthermore, better results with PCSK-9 inhibitor (evolocumab) were observed in the sub-analysis of the post-ACS patients of the FOURIER study when the PCSK-9 inhibitor was started early after the index event compared to beginning the therapy after more than 12 months post-ACS [53]. The first results of the HUYGENS study presented at the ESC annual congress in 2021 showed that the addition of evolocumab to high dose statin therapy leads to increased fibrous cap thickness and fewer lipids in the core of the atherosclerotic plaque, forming a more stable atherosclerotic plaque [54].

Novel lipid lowering therapies are under investigation, e.g., inclisiran [55], anacetrapib [56, 57], evinacumab (ANGPTL3 inhibitor) [58], bempedoic acid [59], pelacarsen [60] but none of them have available data in patients after ACS yet.

Familial hypercholesterolaemia (FH) deserves special attention. Patients with FH have higher levels of LDL-C and higher all-cause and cardiovascular mortality [61]. The prevalence of genetically confirmed FH in patients with ACS below the age of 65 and LDL-C >160 mg/dL (4.14 mmol/L) was 9%. Nevertheless, when using the Dutch Lipid Clinic (DLC) criteria, the prevalence of probable to definite FH increased to 27.2% [62]. Thus, it is essential not to forget to screen for patients with FH in the post-ACS population.

Even after achieving the CV risk factor goals, patients remain at increased cardiovascular risk. One of the possible factors is higher levels of plasmatic triglycerides [63]. Some novel triglyceride-lowering drugs have been shown to decrease triglycerides and atherogenic lipoproteins in those patients with moderate hypertriglyceridemia who are at high risk for or with established cardiovascular disease [64]. Unfortunately, cardiovascular outcome data in post-ACS is lacking.

### 3.3 Renin-Angiotensin-Aldosterone System Inhibitors

In patients with left ventricular (LV) dysfunction, angiotensin-converting enzyme (ACE) inhibitors can lower mortality, AMI and stroke rates [65]. ACE inhibitors (or ARBs – angiotensin receptor blockers when ACE inhibitors are not tolerated) are indicated in post-ACS patients with concomitant LV ejection fraction ≤40%, hypertension, diabetes or renal impairment [15]. The American guidelines recommend an ACE inhibitor for patients with anterior location or heart failure [66].

### 3.4 Beta-Blockers 

Historically beta-blocker use decreased cardiovascular deaths and any subsequent AMI by 30% [67]. In the reperfusion era, beta-blocker therapy reduces the short-term recurrence of AMI and angina but not mortality [68]. Long term beta-blocker usage in patients after ACS without heart failure in the reperfusion era remains controversial [69, 70]. At least two randomised trials testing the efficacy of beta-blocker therapy after myocardial infarction without reduced left ventricular systolic function are ongoing, with results expected by the end of 2022 and 2023 [71, 72].

### 3.5 Diabetes and Glucose-Lowering Drugs 

Even in patients admitted for ACS without known diabetes, the prevalence of disturbances in glucose metabolism is high [73]. An abnormal oral glucose tolerance test upon discharge is a strong risk factor for future cardiovascular events [74]. Nowadays, we have glucose-lowering agents with proven cardioprotective data. In the last guidelines on cardiovascular prevention, using a GLP-1RA or SGLT2 inhibitor with proven outcome benefits is recommended in those with type 2 diabetes mellitus and atherosclerotic cardiovascular disease (ASCVD) to reduce cardiovascular and/or cardiorenal outcomes [50]. Thus screening for diabetes is mandatory in all post-ACS patients.

### 3.6 Vaccination

It has been observed that the incidence of AMI increases during seasonal influenza epidemics [75]. In a meta-analysis, influenza vaccination leads to a 36% reduction of major adverse cardiovascular events in high-risk patients [76]. The administration of an influenza vaccine within 72 hours of a coronary angiography for AMI resulted in a reduction of all-cause mortality (HR 0.59), cardiovascular death (HR 0.59) and myocardial infarction (HR 0.86). The effect of an influenza vaccine administration was observed early after the index event [77]. The protective effect of influenza vaccination is essential in both primary and secondary prevention, and Habib *et al*. [78] called on cardiologists to “promote influenza vaccination and actively advice their patients to get the seasonal influenza vaccination”.

By the end of 2019, the world was unsettled by the COVID-19 pandemic. Patients with underlying cardiovascular diseases have five times higher mortality when encountering the SARS-CoV2 virus [79]. Thus, patients with cardiovascular diseases were among the first to be vaccinated against COVID-19. The ESC advocates the administration of SARS-CoV-2 vaccines to patients with prior cardiovascular diseases [80].

### 3.7 Anti-Inflammatory Treatment

Although the role of inflammation in the initiation and progression of atherosclerosis has been known for a long time [3, 4], only recently have we had studies which prove the beneficial effect of anti-inflammatory therapies on cardiovascular outcomes [81]. A recent meta-analysis showed that the addition of low-dose colchicine to the guideline-recommended treatments in patients with recent AMI or chronic coronary disease reduced the risk of major adverse cardiac events by 25% (RR 0.75) and the risk of myocardial infarction by 22% (RR 0.78), with no effect on all-cause mortality (RR 1.08) but a decreased risk of cardiovascular death (RR 0.82) [82]. Still, the use of colchicine remains reserved for a highly selected population of post-ACS patients, mostly those with recurrent events.

### 3.8 Other

Hyperuricaemia is a well-established risk factor for gout and gout kidney disease. The association between higher uric acid (UA) levels and cardiovascular events is known from the Framingham study. In recent years some studies have shown the potential benefits of UA lowering therapies on cardiovascular outcomes [83]. Currently, we are awaiting the results of the ALL-HEART study which randomized patients with established ischemic heart disease (including post myocardial infarction patients) to a treatment of allopurinol or placebo [83]. At time of publication, the treatment of hyperuricaemia is indicated only as the prevention and treatment of gout.

While patients after STEMI with identifiable standard modifiable cardiovascular risk factors (SMuRFs) are at increased risk of early all-cause mortality, patients without SMuRFs are at risk too, which is even higher than in those with SMuRF [84]. The risk disappeared when taking into account the guideline-recommended secondary prevention pharmacotherapy, underlining the need to treat all STEMI patients with guideline-recommended treatments irrespective of any identified risk factors [84].

## 4. Secondary Prevention Delivery

The challenge is how to get proven secondary prevention measures to the patients. A comprehensive multidisciplinary approach that encompasses all of the aspects mentioned above is needed. Nowadays, such an approach is operated under the cardiac rehabilitation (CR) umbrella [30].

Cardiac rehabilitation “is the sum of activities required to positively influence the underlying causes of the disease, as well as to ensure that patients have the best possible physical, mental, and social conditions, so that, by their efforts, preserve or resume when lost, a place as normal as possible in the life of the community” [85]. In his Presidential Advisory, Balady *et al*. [86] said: “The goal of cardiac rehabilitation and secondary prevention is to stabilise, slow or even reverse the progression of CVD, which in turn reduces the risk of a future cardiac event”. CR offers the opportunity to implement lifestyle changes and is also a time of intensive contact between the patient and the healthcare system and offers the chance to optimise medical treatment post-ACS [87]. Thus the aims of CR are (a) the implementation of a personalised exercise training program, (b) increased control of cardiovascular risk factors, and (c) optimisation of cardiovascular medical treatment [88].

Nowadays, CR is a well-established and evidence-supported approach. To be effective CR, must encompass all the core components as highlighted in the last update of the EAPC position paper (Table 2) [30]. CR delivery depends on local health system resources and historical settings [89].

**Table 2.** **Core components of cardiac rehabilitation/secondary prevention delivery**.

Component	Expected outcomes
Patient assessment	Formulation of personalised objectives of the cardiac rehabilitation programme
Physical activity counselling	Increase participation in physical activity
	Improve psychosocial well-being, aerobic fitness, and prognosis
	Reduce frailty risk
Exercise training	Increase all components of exercise tolerance
	Reduction of symptoms
	Decrease in cardiovascular risk
Diet/Nutritional counselling	Adherence to a healthy diet lowering cardiovascular risk
Weight control management	Maintenance or attainment of a healthy weight
Lipid management	LDL cholesterol under 1.4 mmol/L and reduction by 50% from baseline values
Blood pressure management	Blood pressure <140/90 mmHg, and lower in patients when well tolerated
Smoking cessation	Long-term abstinence from smoking
Psychosocial management	Absence of clinically significant psychosocial problems and acquisition of stress management skills
Evaluation of the program results and establishment of structured follow-up	Quality assurance

Nevertheless, to obtain mortality improvement, CR should be started early (within three months after discharge) after ACS, it must be structured based around adequate exercise volume, be multicomponent and delivered by a multidisciplinary team of qualified health care professionals [90]. As demonstrated by the CROS and CROS-II meta-analysis, such CR programs meeting minimal standards effectively reduce the total mortality beyond modern medication [91, 92]. This evidence is also supported by the secondary analysis of the OMEGA study population of patients after AMI comparing patients who attended comprehensive CR in Germany and those who did not. At 1-year after the index event, patients attending CR had reduced total mortality (OR 0.46) and significant cerebrovascular and cardiovascular events (OR 0.53) [93]. In coronary artery disease, exercise-based CR is an established measure associated with a reduction of the relative risk (RR) of cardiovascular mortality (RR = 0.74), the reduction of hospital admissions (RR = 0.82) and an increase in patient’s quality of life [94].

The recommended exercise training prescription follows the FITT model (frequency, intensity, time and type). As a general recommendation exercise should take place on most days of the week (at least 3 days/week) for aerobic training and 2 times/week for resistance exercises. The exercise intensity should be at least moderate and in selected stable patients can be increased to moderate-to-high intensity. Each session of exercise should last for at least 20–30 minutes (optimally 45–60 minutes). The main types of exercise are aerobic and resistance/strength training, which can be complemented by flexibility training, balance training and inspiratory muscle training [30].

As knowledge of the cardiovascular risk factors is insufficient in patients eligible for secondary prevention measures [95], education programs should be part of comprehensive CR programs.

The prognostic benefits of CR extend beyond the first year. At five years, patients attending the CR program had lower total and cardiovascular mortality as well as lower hospitalisation rates than non-attenders [96]. The GOSPEL trial, a multifactorial, continued reinforced intervention up to 3 years after rehabilitation following AMI, effectively decrease the risk of nonfatal AMI, and a better prescription of drugs for secondary prevention was seen in the intervention group [97].

CR is also effective in medication optimisation. An early follow-up visit after AMI is associated with better short- and long-term adherence to medication [98]. During the METRO study, a real-life investigation of the effect of CR on medication optimisation, a RAS inhibitor optimisation occurred in a third of patients. The same was observed for the alteration of beta-blocker therapy [87]. Medication optimisation was not the only benefit of CR on preventive pharmacotherapy, patients attending CR also had better medication adherence [99].

Although CR effectively delivers secondary preventive measures to post ACS patients, the attendance rates are not optimal [12, 100]. The non-attendance has several risk factors: distance to the CR provider, smoking, a higher burden of comorbidities, and male sex being the most prominent [100]. One possible way to overcome the attendance issue is to automate CR uptake and schedule an early follow-up.

During the COVID-19 pandemic, health care resources were diverted to more acute settings. Nevertheless, CR has retained its importance in patients with cardiovascular disease and with long-COVID [101, 102]. New modes of CR delivery apart from the traditional inpatient and outpatient programmes are envisioned, home-based cardiac rehabilitation overcomes some geographical, logistical and time restricting barriers to CR [103, 104]. Hybrid cardiac rehabilitation is comparable in short-term outcomes to traditional CR [105].

## 5. Audit—Quality Measures

Evaluating the quality of care is an integral part of modern healthcare services. The care process is measured through quality indicators (QIs) or performance measures (PMs). In this context, it has become a widely used practice and an indispensable tool, much sought after by health authorities, the general public, the press and even patients themselves [106].

The American College of Cardiology (ACC) and the AHA have jointly published several documents and position papers where the QIs and PMs of quality of care in the setting of AMI were defined [107]. At first, the optimal methodology for describing and defining QIs and PMs [108, 109], the statistics suitable for public reporting [110] and, the use of composite indicators [111] were published. In 2006, the first set of PMs for AMI was published by the ACC/AHA [106, 112].

In this context, in 2017, the Acute Cardiovascular Care Association (ACVC) of the ESC created the first set of quality indicators, which were in line with the ESC’s recommendations for the management of patients with acute myocardial infarction with or without ST elevation [14, 106]. Three years after the initial set of QIs, an update with accumulated experience and the changes in the supporting evidence were published. As a result, the QIs covered seven care domains: centre organisation, reperfusion/invasive strategies, in-hospital risk assessment, antithrombotic treatment, secondary prevention, patient satisfaction, and outcome and composite quality indicators. Currently, those QIs in the 5th domain which focus on secondary prevention cover the prescribing of three therapeutic classes (statins, angiotensin-converting-enzyme inhibitors (ACEI) or angiotensin II receptor blockers (ARBs) if intolerant of ACEI and beta-blockers). The measurement and reporting of health care performance are associated with better clinical results [113, 114]. In 2020, an assessment of the quality indicators for AMI management from 28 countries and the use of composite quality indicators for benchmarking was published. Data was extracted from the long term follow up of antithrombotic management patterns in acute coronary syndrome patients (EPICOR and EPICOR Asia) registries. Most individual quality indicators were associated with reduced two-year mortality, and their predictive value should receive further attention. Higher compliance using composite quality indicators is associated with lower mortality at the centre, national, and regional levels [115].

## 6. Conclusions

Patients after acute myocardial infarction profit from intensive intervention toward risk factors, from lifestyle through established to novel risk factors. As atherosclerosis and its complications are multidimensional, so secondary prevention is too. Secondary preventive measures after ACS lower CV morbidity and mortality and total mortality, and lastly are much cheaper than acute cardiovascular care. It is thus vital to pay enough attention to getting secondary prevention to as many patients as possible.

## Abbreviations

ACC, American College of Cardiology; ACEI, angiotensin-converting enzyme inhibitor; ACS, acute coronary syndrome; ACVC, The Association for Acute Cardiovascular Care; AHA, American Heart Association; AMI, acute myocardial infarction; ANGPTL3, Angiopoietin-like protein 3; ARBs, Angiotensin II receptor blockers; ASA, acetylsalicylic acid; ASCVD, atherosclerotic cardiovascular disease; CR, cardiac rehabilitation; CV, cardiovascular; CVD, cardiovascular disease; DAPT, dual antiplatelet therapy; DASH, Dietary Approaches to Stop Hypertension; DBP, diastolic blood pressure; DLC, Dutch Lipid Clinic; EAPC, the European Association of Preventive Cardiology; ESC, European Society of Cardiology; FH, familial hypercholesterolaemia; GLP-1RA, glucagon-like peptide 1 receptor agonist; HRQoL, heart-related quality of life; HDL-C, high-density lipoprotein cholesterol; HR, hazard ratio; IHD, ischemic heart disease; LDL-C, low-density lipoprotein cholesterol; LV, left ventricular; MI, myocardial infarction; NSTEMI, non-ST-elevation myocardial infarction; OR, odds ratio; PCSK9, proprotein convertase subtilisin/kexin type 9; PM, performance measure; PSRFs, psychosocial risk factors; QIs, quality indicators; RAAS, renin-angiotensin aldosterone system; RR, risk ratio; SBP, systolic blood pressure; SGLT2, sodium-glucose cotransporter-2; SMuRFs, standard modifiable cardiovascular risk factors; SOS, Swedish Obese Subjects; SP, secondary prevention; STEMI, ST-elevation myocardial infarction; UA, uric acid; US, the United States; USPSTF, the United States Preventive Services Task Force.
